# Imaging glutathione depletion in the rat brain using ascorbate-derived hyperpolarized MR and PET probes

**DOI:** 10.1038/s41598-018-26296-6

**Published:** 2018-05-21

**Authors:** Hecong Qin, Valerie N. Carroll, Renuka Sriram, Javier E. Villanueva-Meyer, Cornelius von Morze, Zhen Jane Wang, Christopher A. Mutch, Kayvan R. Keshari, Robert R. Flavell, John Kurhanewicz, David M. Wilson

**Affiliations:** 1 Department of Radiology and Biomedical Imaging, University of California San Francisco, San Francisco, CA 94143 USA; 2 UC Berkeley – UCSF Graduate Program in Bioengineering, University of California, San Francisco and Berkeley, CA 94143 USA; 3 Department of Radiology, Memorial Sloan Kettering Cancer Center, New York, NY 10065 USA; 4 Molecular Pharmacology Program, Memorial Sloan Kettering Cancer Center, New York, NY 10065 USA; 5 Weill Cornell Medical College, New York, NY 10065 USA

## Abstract

Oxidative stress is a critical feature of several common neurologic disorders. The brain is well adapted to neutralize oxidative injury by maintaining a high steady-state concentration of small-molecule intracellular antioxidants including glutathione in astrocytes and ascorbic acid in neurons. Ascorbate-derived imaging probes for hyperpolarized ^13^C magnetic resonance spectroscopy and positron emission tomography have been used to study redox changes (antioxidant depletion and reactive oxygen species accumulation) *in vivo*. In this study, we applied these imaging probes to the normal rat brain and a rat model of glutathione depletion. We first studied hyperpolarized [1-^13^C]dehydroascorbate in the normal rat brain, demonstrating its robust conversion to [1-^13^C]vitamin C, consistent with rapid transport of the oxidized form across the blood-brain barrier. We next showed that the kinetic rate of this conversion decreased by nearly 50% after glutathione depletion by diethyl maleate treatment. Finally, we showed that dehydroascorbate labeled for positron emission tomography, namely [1-^11^C]dehydroascorbate, showed no change in brain signal accumulation after diethyl maleate treatment. These results suggest that hyperpolarized [1-^13^C]dehydroascorbate may be used to non-invasively detect oxidative stress in common disorders of the brain.

## Introduction

Reactive oxygen species (ROS) are expected products of oxidative metabolism, and must be tightly regulated in the normal brain. Several pathways are required to maintain redox homeostasis, in particular the glutathione-glutaredoxin and thioredoxin-thioredoxin reductase systems^1^. These systems maintain high steady-state concentrations of small-molecule antioxidants including reduced glutathione (GSH) and vitamin C (VitC). Glial cells harbor a high concentration (in the mM range) of GSH, while neurons capture the majority of the VitC in the central neural system^2,3^. Although the reasons for this partitioning are only partially understood, these antioxidants are considered critical in both health and disease. Oxidative stress, marked by increased ROS production and GSH consumption, is implicated in various neurodegenerative and neuropsychiatric disorders such as Parkinson’s disease, Alzheimer’s disease, multiple sclerosis, and schizophrenia^4,5^. Therefore, non-invasive evaluation of brain antioxidants could provide critical insights into these disease processes.

**Figure 1:**
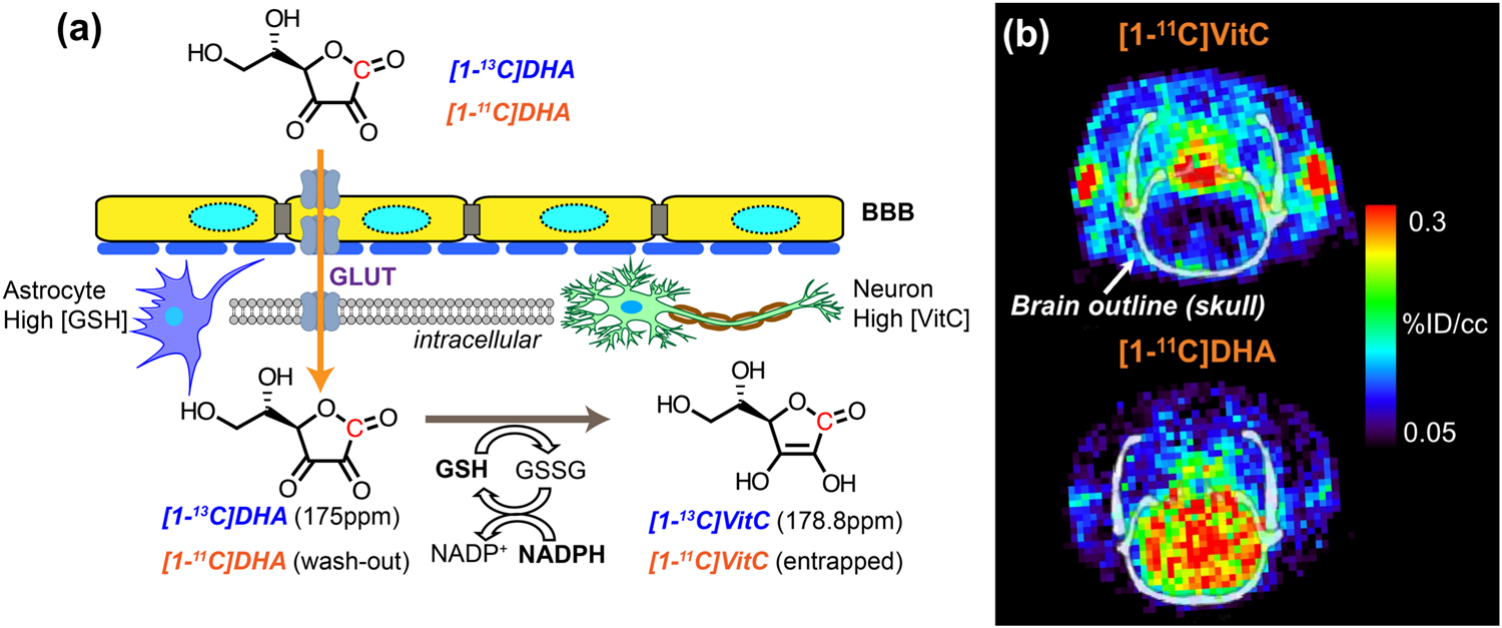
Ascorbates as redox imaging probes. **(a)** Dehydroascorbate (DHA) can traverse the blood-brain-barrier, enter cells via glucose transporter (GLUT)1,3,4 and be rapidly reduced to ascorbic acid (vitamin C, VitC) by intracellular antioxidants including glutathione (GSH), which is coupled with the pentose phosphate pathway via NADPH. The conversion from hyperpolarized [1-^13^C]DHA to [1-^13^C]VitC can be monitored by MR spectroscopy, taking advantage of their unique chemical shifts. The C1 position of DHA and VitC can also be labelled with ^11^C for PET and biodistribution studies. **(b)** In these PET images (adapted from Carroll *et al.*^21^), the accumulation of [1-^11^C]DHA in the normal rat brain is compared to that of [1-^11^C]VitC in the normal rat brain. In normal rats, higher brain signal for [1-^11^C]DHA is expected since it readily crosses the blood-brain barrier and is transported into cells via GLUT transporters. In contrast, transport of VitC into the brain via sodium-dependent vitamin C transporter-2 (SVCT2) is a slower process. Unlike measuring the real-time conversion rate by hyperpolarized MR, PET and biodistribution with [^11^C]ascorbates measures the uptake and retention of ^11^C radiopharmaceuticals.

The relationship between VitC and its oxidized counterpart dehydroascorbic acid (DHA) has been extensively studied. In contrast to VitC, which is slowly imported via sodium-dependent vitamin C transporter-2 (SVCT-2) and maintained at high steady-state concentrations in the brain^6^, DHA is a transient species with remarkably different transport properties despite its structural similarity to VitC. DHA readily crosses the blood-brain barrier (BBB), actively transported by glucose transporters (GLUTs) as shown in Fig. 1a^7–9^. Once transported into cells, DHA undergoes a GSH-dependent, two-electron reduction to VitC, catalyzed by redox enzymes including glutaredoxin, omega-class glutathione transferases and protein-disulfide isomerase^10–13^. This GSH-dependent conversion is one of the motivations for studying ascorbate-derived imaging agents, whose interconversion depends on both the concentration of GSH and the presence or absence of reactive oxygen species^14,15^. In particular, [1-^13^C]DHA, a ^13^C enriched version of dehydroascorbic acid, has been studied with hyperpolarized (HP) ^13^C magnetic resonance (MR) in preclinical models of cancer, diabetes, acute kidney injury, and fatty liver disease^15–20^. In addition, ascorbates labeled for positron emission tomography (PET), namely [1-^11^C]DHA and [1-^11^C]VitC, have recently been synthesized and studied in ROS-producing immune cells *in vitro*^21^. Together, these studies suggest that ascorbate-derived imaging methods may be able to image oxidative stress *in vivo*. In particular, DHA-derived imaging agents were designed to image intracellular redox capacity, typically defined as the concentration of intracellular reducing equivalents and the ability to regenerate them. We therefore compared these methodologies in an animal model of pharmacologic glutathione depletion, to determine if ascorbate-derived HP MR or PET probes could detect the modulation of redox capacity.

Although [1-^13^C]DHA and [1-^11^C]DHA are chemically and biochemically equivalent, the techniques used to study them *in vivo* are considerably different. HP ^13^C MR via dissolution dynamic nuclear polarization (dDNP) is an emerging imaging technology that allows direct visualization of metabolic conversion of ^13^C-enriched molecules, and has been applied extensively to study metabolism in cells, tissues, animal models, and patients^22–24^. dDNP transfers polarization from electron spins to ^13^C nuclei spins at extreme low temperature (0.8–1.3 °K) through microwave irradiation, providing more than 10,000-fold MR signal enhancement of ^13^C-labelled substrates^25^. The HP substrate can be subsequently injected into a living system and its conversion to other metabolites can be monitored by MR spectroscopy in a rapid, real-time manner. HP MR with [1-^13^C]DHA takes advantage of the chemical shift between [1-^13^C]DHA and [1-^13^C]VitC (3.8 ppm) and the drastic signal enhancement (~10% polarization, >10^4^-fold at 3 Tesla) provided by dDNP to enable *in vivo* interrogation of redox capacity. Since the hyperpolarized signal lifetime is prescribed by the T_1_ of the substrate (approximately 57 s for [1-^13^C]DHA at 3 Tesla), HP ^13^C signal can be observed *in vivo* for approximately 1 to 2 minutes at most^23^. Therefore, the real-time detection of [1-^13^C]DHA to [1-^13^C]VitC *in vivo* is primarily a kinetic measurement that depends on substrate transport and the availability of intracellular reducing equivalents.

In contrast, PET is an established metabolic imaging technology whereby a positron-emitting isotope, such as ^11^C or ^18^F, is incorporated into a drug or metabolite of interest, and usually imaged following a significant delay depending on the half-life of the radioactive isotope. Therefore, the primary determinants of image contrast are the transport and retention/entrapment of the radiopharmaceutical, and the clearance of nonspecific signal. As shown in Fig. 1b, PET images of rat brains demonstrated the distinctive difference in accumulated signal between [1-^11^C]DHA and [1-^11^C]VitC, as previously reported, reflecting their differential transport across the blood-brain barrier and cellular membrane^21^. In the case of [1-^11^C]DHA, the radiopharmaceutical is expected to be entrapped inside the cell once it is reduced to [1-^11^C]VitC, a charged molecule at physiological pH, and unreacted [1-^11^C]DHA may be washed out. We hypothesized that retention of [1-^11^C]DHA would depend partially on intracellular reducing agents including GSH^26^, and therefore may also be modulated following glutathione-depletion.

In this study, we aimed to characterize ascorbate-derived HP ^13^C MR and ^11^C PET probes for brain imaging, and take advantage of their different capabilities to assess *in vivo* redox capacity of the rat brain. First, we studied the transport, compartmentalization, and biodistribution of both DHA and VitC in normal rat brain with HP ^13^C MR and ^11^C PET probes. Then we modulated GSH content in rats by pharmacological treatment and studied the changes in DHA to VitC conversion in the brain using both HP ^13^C MR and ^11^C PET probes. Specifically, four groups of Sprague-Dawley rats were studied using: (1) two-dimensional (2D) chemical shift imaging (CSI) with HP [1-^13^C]DHA and [1-^13^C]VitC separately to determine the transport and compartmentalization of ascorbates in the head region (n = 6 for DHA, n = 3 for VitC); (2) one-dimensional (1D) slice-selective dynamic spectroscopy with HP [1-^13^C]DHA, at 24 hours before and 2 hours after diethyl maleate (DEM) intraperitoneal injection (4.6 mmol/kg)^27^, to measure the real-time DHA to VitC conversion rate (n = 4); (3) biodistribution using [1-^11^C]DHA and [1-^11^C]VitC, respectively, to determine the accumulation of ascorbate-derived probes in the brain and other organs (n = 3 each); (4) biodistribution with [1-^11^C]DHA in rats with DEM treatment as in group (2) to measure ^11^C probe accumulation following glutathione depletion (n = 3). Additionally, 1D slice-selective dynamic spectroscopy with HP [1-^13^C]DHA at 60% concentration of group (2) was performed to demonstrate DHA to VitC reaction order (n = 3), and an *in vitro* GSH assay was performed on brain tissues collected from normal and DEM-treated rats to validate the redox modulation by DEM (n = 7 each).

## Results

### Behavior of HP ^13^C DHA and VitC *in vivo* is consistent with known features of blood-brain barrier penetration and transport

**Figure 2:**
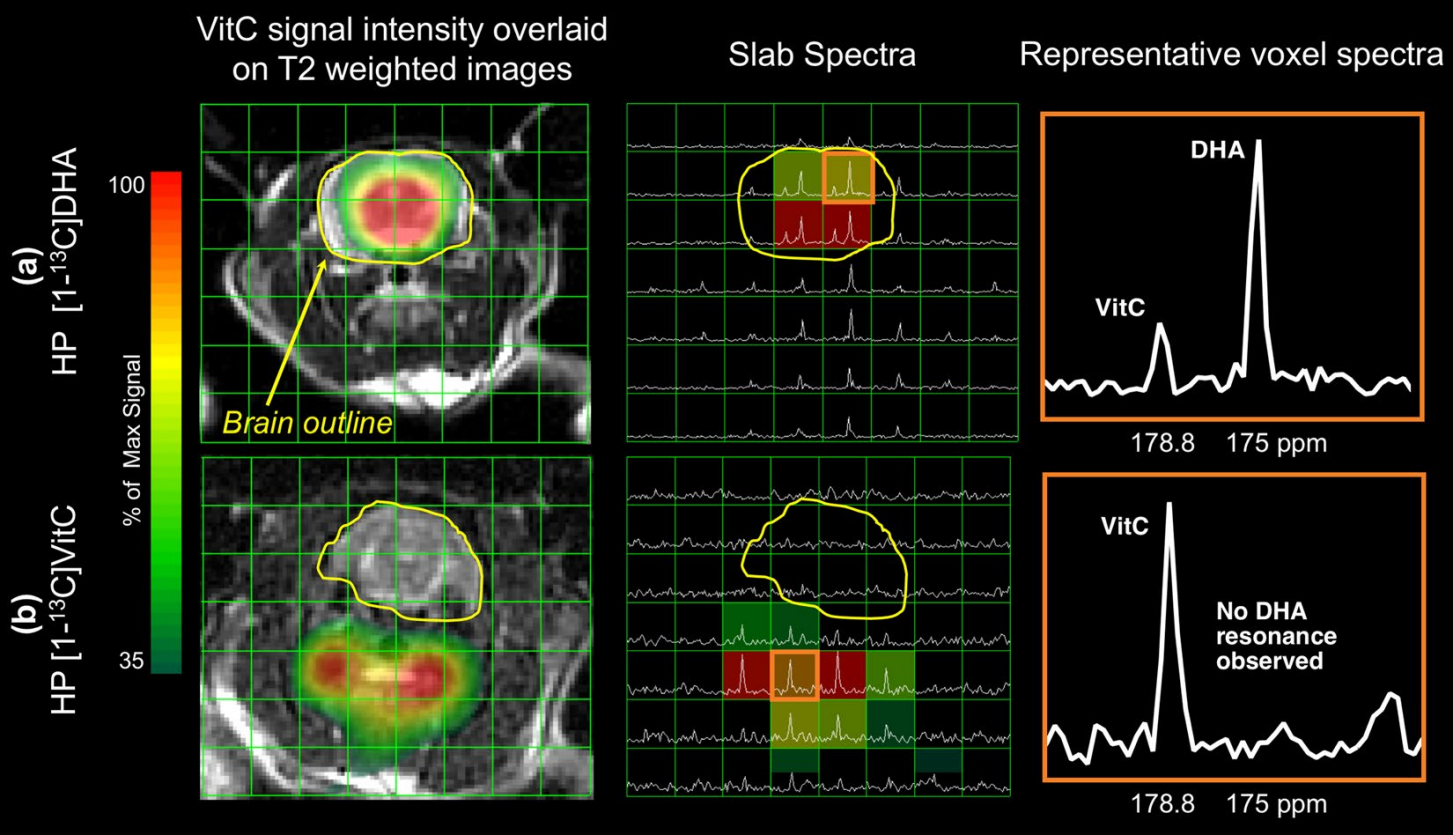
2D Chemical Shift Imaging with hyperpolarized ^13^C ascorbates reveals different transport and compartmentalization of dehydroascorbate (DHA) and vitamin C (VitC). (**a**) Injection of hyperpolarized [1-^13^C]DHA, with conversion to VitC in the normal rat brain. The VitC resonance is only observed in voxels corresponding to the brain as shown in the VitC signal intensity map and slab spectra, while the DHA resonance is observed both in the brain and surrounding tissue. A representative voxel corresponding to brain tissue shows both a resonance corresponding to the introduced hyperpolarized [1-^13^C]DHA and its metabolite [1-^13^C]VitC. (**b**) Injection of hyperpolarized [1-^13^C]VitC in normal rats. Lower (in this case negligible) VitC signal is seen in voxels corresponding to brain as shown in the VitC signal intensity map and slab spectra, and no oxidation to DHA is observed. A representative voxel corresponding to tissues in the neck show a resonance corresponding to the introduced hyperpolarized [1-^13^C]VitC without evidence of metabolic conversion.

Both HP [1-^13^C]DHA and HP [1-^13^C]VitC were administered separately to normal rats (group 1). Following HP [1-^13^C]DHA injection, its reduction to [1-^13^C]VitC was observed in the brain, demonstrated by the presence of a new downfield resonance (approximate 3.8ppm) in the ^13^C MR spectrum (n = 6). For HP [1-^13^C]DHA study, the DHA resonance was observed in most tissue voxels including brain and its surrounding tissues (muscle and vasculature), while the VitC metabolite was only observed in the brain voxels (Fig. 2a) with VitC/(DHA + VitC) = 0.48 ± 0.15 (estimated from 18 brain voxels), consistent with penetration of DHA across the blood-brain barrier. In contrast, when HP VitC is injected, the 2D CSI (n = 3) found drastically lower VitC signal in the brain (mostly below quantification limit) than in surrounding tissues and no oxidation to DHA (Fig. 2b). These data are consistent with the slower transport of VitC across the blood-brain barrier, and the high reducing capacity of the normal brain^8^.

### Glutathione depletion via diethyl maleate results in a lower rate of DHA-to-VitC conversion

**Figure 3:**
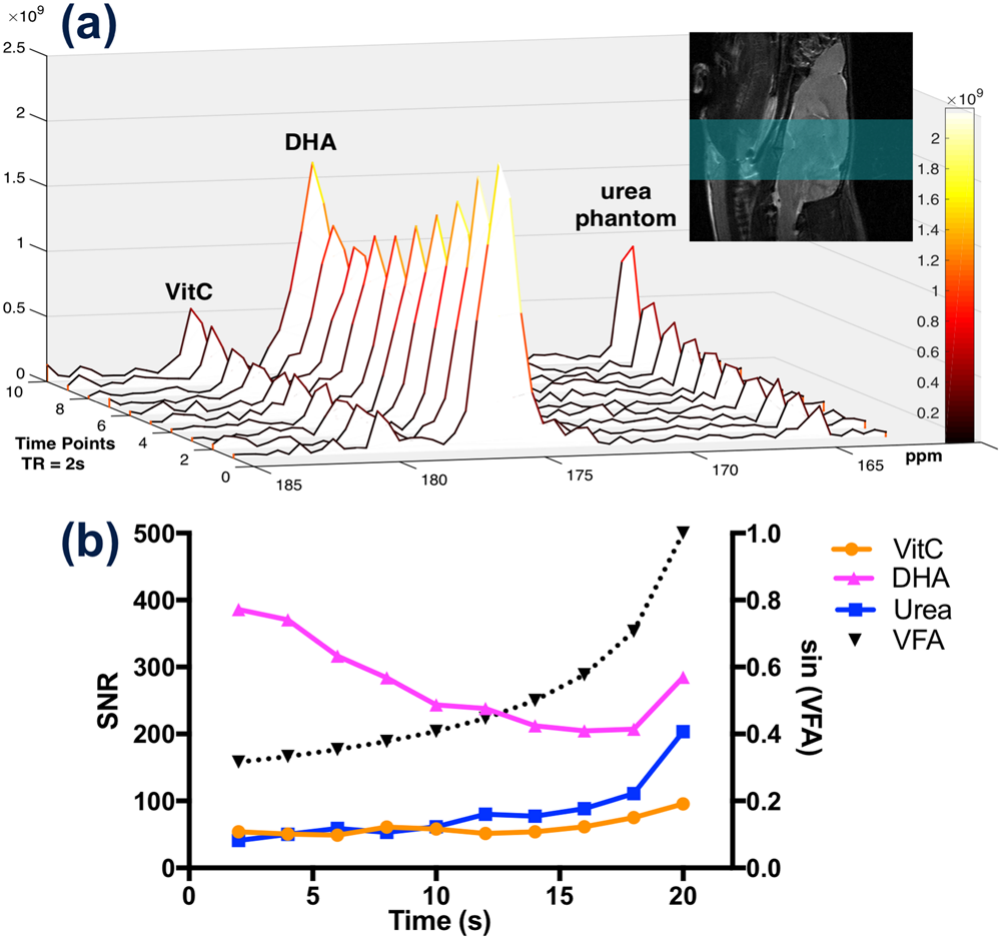
Dynamic 1D slice-selective spectroscopy reveals real-time hyperpolarized [1-^13^C]dehydroascorbate (DHA) to [1-^13^C]vitamin C (VitC) conversion. MR spectra of a normal rat brain is shown in (**a**), and signal-to-noise ratio quantification of DHA, VitC, and urea phantom (solid line), as well as variable flip angle (VFA) scheme (dash line), are plotted in (**b**). Hyperpolarized [1-^13^C]VitC signal in a normal rat brain gradually increases over time despite the rapid longitudinal relaxation (*in vitro* T_1_ ~ 29.2 s at 3 Tesla), and hyperpolarized [1-^13^C]DHA signal gradually decreases due to metabolic conversion and longitudinal relaxation (*in vitro* T_1_ ~ 56.5 s at 3 Tesla). Signal corresponding to the [^13^C]urea phantom (placed next to the rat head) also gradually increases, demonstrating the VFA scheme. A sagittal T_2_-weighted image demonstrates the imaging slab (green highlighted region).

Using a 1D dynamic HP [1-^13^C]DHA study, we investigated whether the DHA to VitC conversion rate is correlated with GSH content in the rat brain (group 2). We adopted a variable flip angle scheme to account for the non-renewable nature of hyperpolarized signal and to utilize signal-to-noise ratio (SNR) efficiently since the T_1_ of [1-^13^C]VitC is relatively short (29.1 s, *in vitro* at 3 T)^16^. Dynamic spectra of the rat brain showed that the [1-^13^C]VitC signal gradually increases during the acquisition window used (Fig. 3), suggesting VitC production rate is greater than signal decay rate mediated by T_1_ relaxation. Because VitC signal is mainly from in the brain while DHA signal is from both the brain and surrounding tissues in the 1D slab spectra, we focused our analysis on the kinetic rates of VitC production as opposed to including both DHA and VitC in the kinetic model or using ratiometric approach.

**Figure 4:**
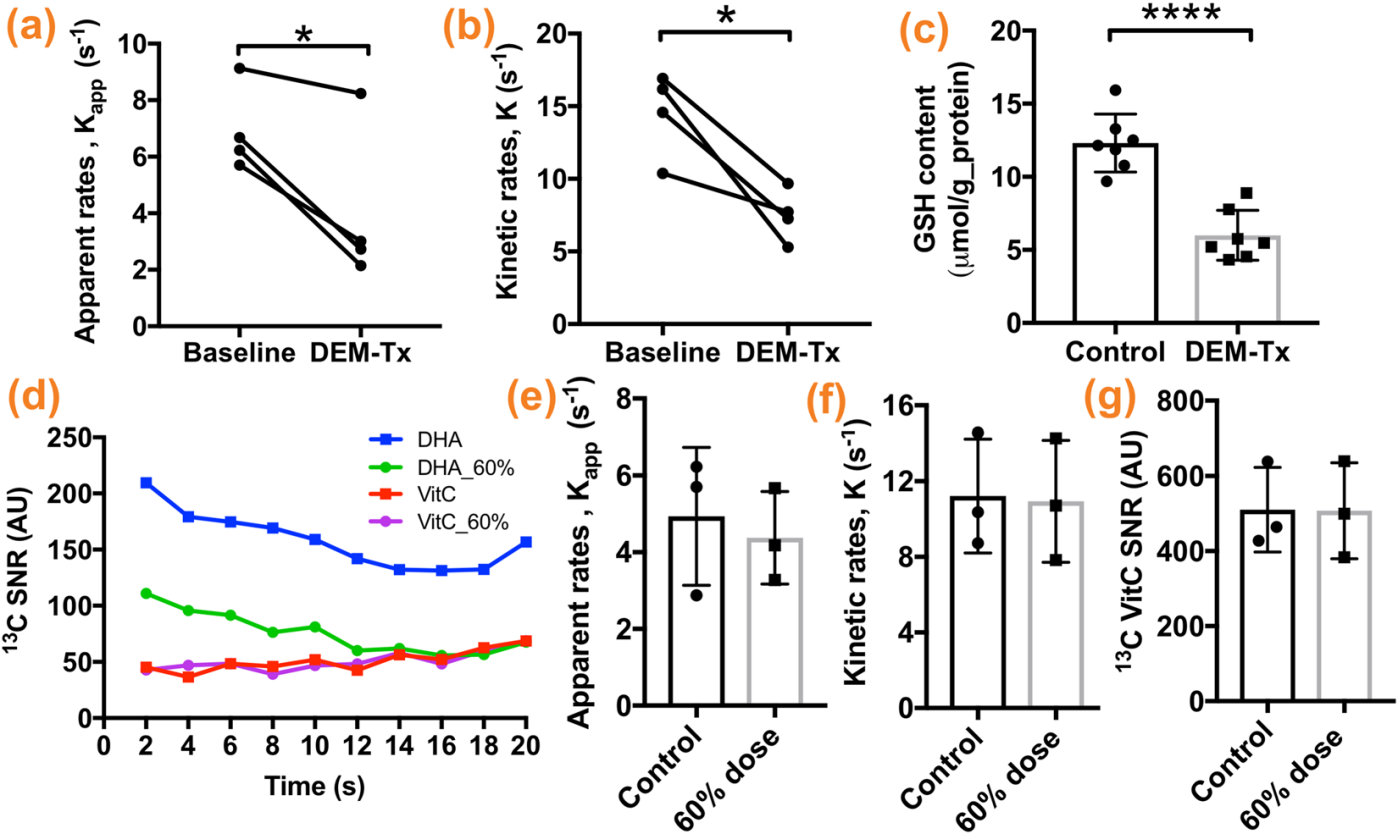
Glutathione depletion via diethyl maleate (DEM) results in a significantly lower rate of [1-^13^C]dehydroascorbate (DHA) to [1-^13^C]vitamin C (VitC) conversion. Both apparent VitC production rate (k_app_) and kinetic rate (k) of VitC production in the brain decreased significantly after DEM treatment (**a**,**b**), consistent with significantly lower brain GSH content in DEM-treated rats compared to control rats (**c**). To demonstrate DHA to VitC reaction order, 1D spectroscopy was performed on a separate cohort of rats with HP [1-^13^C]DHA at 60% of the usual dose. The average signal of [1-^13^C]DHA and [1-^13^C]VitC at each time point were plotted (**d**), and VitC signal appears unchanged with decreased DHA dose. The resulting k_app_, k, and total ^13^C VitC SNR are not significantly different from the control group (**e**,**f**,**g**). (*p < 0.05, ****p < 0.0001).

We found the apparent VitC production rate (k_app_) decreased by nearly 40% from 6.94 ± 1.52 s^−1^ to 4.03 ± 2.83 s^−1^ (p = 0.0293) after DEM treatment (Fig. 4a). The apparent *in vivo* T_1_ of VitC was estimated to be 7.80 ± 1.28 s (n = 9) using a subset of data (3 datasets were excluded due to poor fitting quality), and we found kinetic rate (k) decreased by nearly 50% from 14.15 ± 2.93 s^−1^ to 7.48 ± 1.80 s^−1^ (p = 0.0218) after DEM treatment (Fig. 4b). Correspondingly, the *in vitro* GSH assay (methods reported in the supplementary information) showed that brain GSH content in the DEM-treated rats is significantly lower than in the control rats (5.99 ± 0.64 vs. 12.31 ± 0.75 μmol/g protein, n = 7, p < 0.0001) (Fig. 4c).

To validate our kinetic modeling of the DHA to VitC conversion, dynamic 1D spectroscopy was performed on a separate cohort of rats (n = 3) with 18 μmoles HP DHA (approximately 60% dose of the control/baseline group). We found there was no statistically significant difference between the control/baseline group and lower dose group in k_app_ (4.94 ± 1.04 vs. 4.38 ± 0.70 s^−1^, p = 0.678), or k (11.22 ± 1.74 vs. 0.94 ± 1.86 s^−1^, p = 0.917) as well as the sum of VitC signal (510.3 ± 65.06 vs. 507.6 ± 73.94 A.U., p = 0.9792) (Fig. 4d–g), suggesting that DHA dose used in our experimental set-up was in excess and not a rate limiting factor for VitC production.

### ^11^C Biodistribution studies showed expected differences between [1-^11^C]DHA and [1-^11^C]VitC, but no effect of diethyl maleate treatment on [1-^11^C]DHA brain accumulation

**Figure 5:**
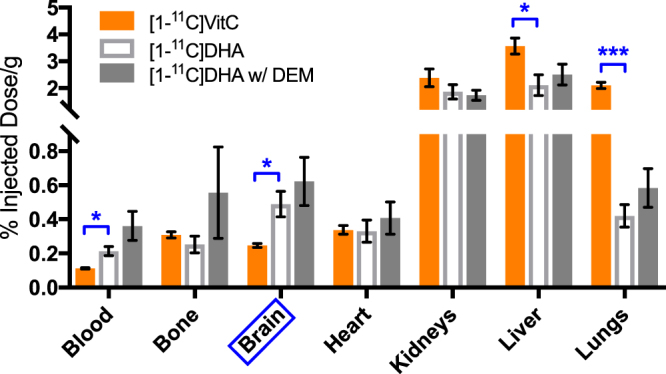
Biodistribution studies reveal different accumulation of [1-^11^C]dehydroascorbate (DHA) and [1-^11^C]vitamin C (VitC) in normal rat brain, but no effect of diethyl maleate (DEM) treatment. Gamma counting of harvested brains showed a significantly higher signal accumulation for [1-^11^C]DHA than [1-^11^C]VitC in the normal brain 1 hour following radiopharmaceutical administration. In addition, there was significantly higher uptake/retention for [1-^11^C]VitC versus [1-^11^C]DHA in the lungs and liver in normal rats. In the DEM-treated rats with [1-^11^C]DHA, no significant differences were found in ^11^C radiopharmaceutical uptake/retention between normal and DEM-treated rats in major organs, including the brain. (*p < 0.05, ***p < 0.001).

The biodistribution of [1-^11^C]DHA or [1-^11^C]VitC was compared by *ex vivo* biodistribution analysis on harvested normal rat tissues 1 hour following administration (group 3, n = 3 each). In addition, [1-^11^C]DHA was administrated to DEM-treated rats (group 4, n = 3) to study the effect of GSH content on ^11^C radiopharmaceutical accumulation. Biodistribution of ^11^C radiopharmaceutical in three groups of rats are shown in Fig. 5. [1-^11^C]VitC showed lower uptake/retention in the brain than [1-^11^C]DHA (0.25 ± 0.012 vs. 0.49 ± 0.075% Injection Dose(ID)/g, p = 0.0330), consistent with our HP MR observations and our previously reported PET imaging study (Fig. 1b). However, lung and liver showed significantly higher uptake/retention for [1-^11^C]VitC than [1-^11^C]DHA (lungs: 2.10 ± 0.12 vs. 0.42 ± 0.066%ID/g, p = 0.0002; liver: 3.57 ± 0.30 vs. 2.12 ± 0.39%ID/g, p = 0.0408). For the glutathione modulation study using [1-^11^C]DHA, no significant difference was found in radiopharmaceutical accumulation for major organs between control and DEM-treated subjects (0.49 ± 0.075 vs. 0.62 ± 0.14%ID/g, p = 0.449).

## Discussion

Non-invasive *in vivo* evaluation of brain redox capacity and oxidative stress could provide insights into neurological disease progression and therapeutic response. In this study, we used ascorbate-based imaging probes to study brain redox capacity leveraging both HP-MR and PET modalities. We first demonstrated that HP [1-^13^C]DHA is readily converted to HP [1-^13^C]VitC in the normal brain, evidenced by 2D chemical shift imaging revealing both DHA and VitC resonances in voxels corresponding to brain tissues following [1-^13^C]DHA injection. This finding is consistent with the penetration of DHA through BBB and its rapid entry into cells. In contrast, when HP [1-^13^C]VitC was administered, lower hyperpolarized signal was seen in brain voxels and no oxidation to HP [1-^13^C]DHA was observed. These findings reflect the slow cellular transport process of VitC via SVCT2 against a concentration gradient, and the role of VitC as an antioxidant reservoir that is not easily oxidized. These features represent the major shortcomings of [1-^13^C]VitC as a HP MR probe for imaging the brain.

After characterizing the transport and compartmentalization of DHA and VitC, we modulated GSH content with DEM treatment which depletes GSH by direct conjugation^27^, and studied the changes in DHA to VitC kinetic rates using dynamic HP ^13^C MR spectroscopy. 2D CSI with radiometric quantification has lower reproducibility due to sensitivity to small differences in acquisition timing and substrate delivery, demonstrated by the variabilities in VitC/DHA ratios in 6 normal rats (reported in Supporting Information). Therefore, we adopted 1D slab dynamic acquisition approach with variable flip angle scheme for efficient SNR usage, which is important given the short T_1_ of [1-^13^C]VitC, to study the effect of redox modulation. In dynamic spectra of normal brain, there was observable real-time production of VitC; we also observed that DHA signal is about 5 times higher than VitC signal, suggesting that cellular transport and/or enzyme and cofactor turnover rate are likely rate-limiting factors for the observed VitC production. In other words, these data suggested that the DHA dose was in excess and not a rate-limiting factor in our experimental set-up. To demonstrate the reaction order, we performed dynamic spectroscopy with lower (60%) doses of [1-^13^C]DHA and found that the kinetic rates of VitC production and sum of VitC SNR are independent of DHA concentration, which validated the pseudo-zero-order kinetic model. To quantify *in vivo* reaction kinetics, we generated two kinetic parameters (reported in Methods section): apparent VitC production rate (k_app_) is a straightforward parameter that reflects the rate of product accumulation, assuming T_1_ relaxation equally affects the control and DEM-treated groups; whereas kinetic rate (k) was generated by non-linear fitting and takes account of T_1_ relaxation. We found that DHA to VitC conversion rate correlated with the brain GSH content, as evidenced by significantly reduced kinetic parameters (k_app_ and k) after DEM treatment that depletes GSH by more than 50%.

There are several important considerations and limitations of our dynamic [1-^13^C]DHA MR study. First, each animal was subjected to two intravenous injections of [1-^13^C]DHA, an oxidant that could deplete GSH, 24 hours apart between baseline and post-DEM-treatment imaging^12^. However, Weber *et al*. have demonstrated that rat brain GSH content returns to normal 24 hours after DEM treatment, a prototypical GSH deleting agent that directly conjugates with GSH and is more potent than DHA^27^. Furthermore, depletion of GSH by DHA could induce increases in other intracellular reducing equivalents (such as thioredoxins) whose pool size would be expected to impact the rate of DHA to VitC reduction. Timm *et al*. have shown that DHA administration resulted in a rapid increase in GSSG/GSH ratio and pentose phosphate pathway (PPP) flux in cells and tumors^20^. It can be argued that the initial DHA administration for baseline imaging could upregulate PPP flux and other reducing equivalents, which would be a confounding factor for our study. Nevertheless, the DEM treatment seemed to offset the increase in PPP flux and other reducing equivalents as the VitC production rate decreased significantly. Second, the supraphysiologic concentration of administered HP [1-^13^C]DHA could saturate the transporter or relevant enzymes and create a rate-limiting condition, or result in substrate inhibition and decrease of enzymatic flux. Third, unlike the first-order reaction model, the kinetic parameters generated from pseudo-zero-order model are highly influenced by the numerical scale (i.e. SNR), which could potentially explain the inter-subject variability. For this reason, our dynamic acquisition and analysis scheme could only detect *the relative changes* in GSH content, but not the absolute concentration.

Another important consideration is the physiologic effect of DEM treatment in rats. We noted that rats became lethargic after DEM treatment. Numerous studies have explored the potential toxic effects of DEM in the heart and lung, and the dose we used was approximately 40% of the LD_50_ reported for oral administration to rats^28–31^. We also observed that the total ^13^C SNR was approximately 35% lower on average in DEM-treated than normal rats despite similar DNP solid state buildup, suggesting less HP ^13^C substrate was delivered to the imaging slab. Although we have demonstrated that the rate of VitC production is independent of DHA dose administered in our supraphysiologic dose range, it is still possible that lower DHA substrate delivery contributed to the lower VitC production rate.

In addition to ascorbate-derived probes designed for HP MR, we also leverage ^11^C radiopharmaceuticals, namely [1-^11^C]DHA and [1-^11^C]VitC, to study the uptake and biodistribution of ascorbates. [1-^11^C]VitC showed lower accumulation than [1-^11^C]DHA in the brain, consistent with HP ^13^C MR findings and previously reported PET imaging data^21^, but higher retention in the lung and liver 1 hour after administration, possibly due to high expression of sodium-depended vitamin C transporters (SVCT) in these tissues^32–34^. [1-^11^C]DHA reduction to [1-^11^C]VitC represents a potential trapping mechanism^21,26^, with unreduced [1-^11^C]DHA likely washed out of the cell. In the DEM redox modulation study using [1-^11^C]DHA, we initially hypothesized that the lower brain GSH content in DEM-treated cannot adequately reduce [1-^11^C]DHA to [1-^11^C]VitC, and un-reacted [1-^11^C]DHA would be washed out resulting less [1-^11^C]VitC retention (lower gamma counting). However, there was no difference in ^11^C radiopharmaceutical retention rate in major organs between the normal and DEM-treated groups.

There are several important differences between HP ^13^C MR and ^11^C biodistribution studies that could explain the inconsistency between their findings. As discussed in Methods section, ^11^C radiopharmaceuticals were administrated at a considerably lower pharmacologic dose compared to HP ^13^C probes (0.16μmol vs. 30μmol). It is possible that residual GSH in DEM-treated rats is adequate to reduce the lower pharmacologic dose of DHA used in ^11^C studies. More importantly, the two technologies differ significantly in their mechanisms of generating image contrast and timing, making direct comparison of data difficult. Biodistribution studies were performed at 1 hour after DHA administration as compared to the 1 minute kinetic analysis applied to HP MR studies. Hence, it appears that although [1-^11^C]VitC was likely generated at a slower rate in GSH-depleted animals, there was insufficient washout of unreacted [1-^11^C]DHA to yield observable differences in ^11^C accumulation. It is possible that dynamic PET imaging or biodistribution studies by gamma counting at earlier time points could reveal subtle differences in the radiopharmaceutical accumulation rate in the brain. Overall, our results suggested that the [1-^11^C]DHA was insensitive to changes in redox capacity in this pharmacologic GSH depletion model using our experiment set-up. This highlights the potential value in studying real-time substrate conversion by HP MR as opposed to probe retention rate by biodistribution of radiopharmaceutical or PET imaging analysis.

In conclusion, we demonstrated the transport and biodistribution features of DHA and VitC in the brain by both HP ^13^C MR and ^11^C PET probes, and found that the kinetic rates of [1-^13^C]DHA to [1-^13^C]VitC conversion correlated with brain GSH content. These studies suggest that hyperpolarized [1-^13^C]DHA can assess brain redox capacity non-invasively, and potentially allow visualization of oxidative stress in numerous brain pathologies.

## Methods

### Dynamic Nuclear Polarization (DNP)

Both [1-^13^C]DHA and [1-^13^C]VitC were prepared, polarized, and dissolved in aqueous media as previously described^14^. A 2.2 M solution of [1-^13^C]DHA dimer (Sigma Aldrich ISOTEC, Miamisburg, OH) in dimethyacetamide (DMA) containing 15 mM OX063 trityl radical (GE Healthcare, Menlo Park, CA) was polarized on a HyperSense DNP instrument (Oxford Instruments, Abingdon, UK) operating at 3.35 Tesla and 1.3°K, achieving approximately 10% polarization after 1 hour microwave irradiation (determined by separate NMR studies using the same material). The frozen sample was then rapidly dissolved in distilled water containing 0.3 mM ethylenediaminetetracetic acid (EDTA) with a final concentration of 15–22 mM and pH of 5.5. For all *in vivo* studies, approximately 30 μmoles of [1-^13^C]DHA (1.3–2.0 ml) were administered intravenously to rats. Similarly, a 2.2 M solution of [1-^13^C]VitC (Omicron Biochemicals, South Bend, IN) was prepared as a sodium salt in NaOH/water/DMSO, polarized, and dissolved in 100 mM phosphate buffer to a final pH of 7.0. For 2D CSI studies, approximately 100 μmoles of HP [1-^13^C]VitC were administered intravenously.

### ^11^C radiopharmaceutical synthesis

[1-^11^C]VitC were synthesized from L-xylosone and [^11^C]HCN precursors using a GE PETtrace cyclotron at our on-site radiopharmaceutical facility as previously described^21^ and reported in the supplementary information. Overall, [1-^11^C]VitC was obtained with a decay-corrected radiochemical yield of 35.8 ± 18% and a specific activity of 1.7 ± 0.4 mCi/μmol (n = 3). [1-^11^C]DHA was obtained by oxidation of [1-^11^C]VitC with a decay-corrected radiochemical yield of 25.8 ± 2.6% and a specific activity of 1.85 ± 0.5 mCi/μmol (n = 6).

### MR studies

All animal experiments were approved by our Institutional Animal Care and Use Committee (IACUC) and performed in accordance with IACUC protocols. Rats were placed under 1.5–2% isoflurane anesthesia and administered 30 μmoles HP [1-^13^C]DHA or 100 μmoles HP [1-^13^C]VitC solution via tail-vein catheter for both 2D CSI studies and dynamic 1D spectroscopy. For group (1) rats (n = 6 for DHA and n = 3 for VitC), two-dimensional single time-point CSI was performed on a GE Signa 3 Tesla MR scanner using a dual-tuned ^1^H-^13^C coil with the following parameters: voxel size = 5 × 5 × 20 mm, TE/TR = 135/210 ms, flip angle = 20°, spectral bandwidth/resolution = 5000/9.77 Hz, and 25 s acquisition delay after the start of injection. For group (2) rats, one-dimensional slice-selective dynamic spectroscopy with HP [1-^13^C]DHA was performed using a similar set-up on a GE Signa 3 Telsa (n = 2 before and after DEM treatment) and a Bruker Biospec 3 Tesla MR scanner (n = 3 before DEM treatment, n = 2 after DEM treatment, n = 3 for 60% dose experiments), with the following parameters: spectral bandwidth/resolution = 25 kHz/12.2 Hz, slice thickness = 10 mm, TR = 2 s, 10 repetitions, RF-compensated variable flip angle scheme^35,36^, and 25 s acquisition delay after the start of injection. Axial and sagittal T_2_-weighted anatomic images were acquired with a fast spin echo sequence prior to HP ^13^C studies and used to prescribe the spectroscopy slab. Rats were fasted for 8 hours before HP ^13^C MR studies to reduce glucose competition with DHA for GLUTs as previously reported^14,17,37^.

### ^11^C biodistribution studies

Rats were placed under 1.5–2% isoflurane anesthesia and administered 304 ± 78 μCi [1-^11^C]DHA or 360 ± 180  μCi [1-^11^C]VitC via tail vein catheters (for group 3 and 4 rats). Animals were subsequently sacrificed at 1 hour after radiopharmaceutical injection, and internalized radioactivity was immediately measured on their harvested organs using a Hidex automatic gamma counter (Turku, Finland) (n = 3 each).

### Data analysis

2D CSI data was reconstructed and overlaid on axial anatomic images using SIVIC^38^; the peak heights of DHA and VitC resonances were manually quantified on the magnitude spectrum. The metabolite ratio, VitC/(DHA + VitC), was calculated for each voxel. For 1D dynamic MR spectroscopy data, SNRs of DHA and VitC resonances for each time point were quantified based on the fit peak integral in MestreNova12 (Madrid, Spain), and were subsequently used to generate kinetic parameters in MATLAB 2017b (Natick, MA). We demonstrated that the DHA to VitC conversion in this experiment was a pseudo-zero-order reaction as shown in Results section, and generated two kinetic parameters based on a zero-order kinetic model assuming no back conversion from VitC to DHA: (1) *apparent VitC production rate* (k_app_) via linear fitting of VitC signal and normalized to total VitC SNR, and (2) *kinetic rate* (k) via non-linear least square fitting of VitC signal with the Trust-Region algorithm to the following equations:

1 $$\frac{d{M}_{z}}{dt}={k}+\frac{1}{{T}_{1}}{M}_{z}$$

2 $${M}_{z,n}=\frac{{M}_{xy,n}}{\sin ({\theta }_{n}){\prod }_{i=1}^{n-1}\,\cos ({\theta }_{i})}$$

where M_z_ is longitudinal magnetization, M_xy_ is transverse magnetization (observed signal), n is the index of time points, and ${\theta }_{n}=\arctan (1/\sqrt{N-n})\,\,$is the flip angle. Dynamic data was first fit into kinetic models with T_1_ as an unknown parameter to estimate apparent *in vivo* T_1_ of VitC, which was subsequently incorporated into the kinetic model as a fixed parameter to generate k assuming T_1_ is the same across the subjects. Apparent kinetic rate **(**k_app_**)** reflects the rate of observed VitC signal change caused by both T_1_ relaxation and VitC production from DHA, while k reflects only the VitC production rate.

A paired Student’s t-test was used to compare kinetic parameters before and after DEM treatment. An unpaired Student’s t-test was used to compare the GSH content of rat brains with and without DEM treatment and biodistribution of [1-^11^C]ascorbates. All statistical analyses were performed using GraphPad Prism 7d (La Jolla, CA). All quantitative results are reported and graphed as mean ± standard deviation; p values < 0.05 were considered statistically significant, and our p values for biodistribution analysis were not adjusted for multiple comparisons.

### Data availability

The datasets generated during and/or analyzed during the current study are available from the corresponding author on reasonable request.

## Electronic supplementary material

Supplementary Information
