# Supplementary material for: Imaging glutathione depletion in the rat brain using ascorbate-derived hyperpolarized MR and PET probes
Source: Sci Rep. 2018 May 21;8:7928. doi: 10.1038/s41598-018-26296-6 (PMC5962598; doi:10.1038/s41598-018-26296-6)
Supplement: Supplementary file 1 — Supplementary Information [file 41598_2018_26296_MOESM1_ESM.docx]

**Imaging glutathione depletion in the rat brain using ascorbate-derived hyperpolarized MR and PET probes**

Hecong Qin, Valerie N. Carroll, Renuka Sriram, Javier E. Villanueva-Meyer, Cornelius von Morze, Zhen Jane Wang, Christopher A. Mutch, Kayvan R. Keshari, Robert R. Flavell, John Kurhanewicz, and David M. Wilson

**Supplementary Information**

***[1-^11^C]VitC Synthesis***

[1-^11^C]VitC was synthesized as previously reported (Carroll et al. 2016). Briefly, 200 mCi [^11^C]KCN was trapped on a 1 mL Teflon tube (OD = 1.35 mm) coated with a minimal volume of 0.5 M KOH. Next vacuum (~630 Torr) was applied to the trap for 10 minutes to remove excess NH_3_ and the line was flushed with N_2_. The activity was eluted from the trap with a 1 mL solution of 1 mg/mL KCN into a vial containing 200 μL of 0.2 M L-xylosone. The resulting imine intermediate was then hydrolyzed with addition of 15 µL 12 M HCl and heated for 15 - 20 minutes at 150°C to yield the desired product. [1-^11^C]VitC was isolated in >99% radiochemical purity by HPLC using a semiprep Hydro-RP column with 4 mL/min 0.1% H_3_PO_4_ isocratic with a t_R_= 7.0 min (Figure 1). The radio peak was collected and confirmed by co-injection with a vitamin C standard (Figure 2) using an analytical HydroRP column, 0.6 mL/min 0.1% H_3_PO_4_ isocratic with a t_R_= 3.6 min. [1-^11^C]VitC was obtained in 35.8 ± 18% radiochemical yield with a specific activity of 1.7 ± 0.4 mCi/μmol (n=3).

Supplemental Figure 1: HPLC isolation of [1-^11^C]VitC using a semiprep Hydro-RP column. Radiochemical peak t_R_ = 7.0 min collected

Supplemental Figure 2: Analytical HPLC showing coinjection of [1-^11^C]VitC with non-radioactive standard

***[1-^11^C]DHA Preparation***

2.5 mL of HPLC purified [1-^11^C]VitC was added to a vial containing 2.25 mg activated charcoal. [1-^11^C]VitC was oxidized with rapid bubbling of O_2_ for 10 minutes. Oxidation was monitored HPLC using an analytical Hydro-RP column with isocratic elution using 0.6 mL/min 0.1% H_3_PO_4_ t_R_ = 4.5 min (Figure 3). The mixture was filtered and used without further purification. [1-^11^C]DHA was obtained in 25.8 ± 2.6% radiochemical yield with a specific activity of 1.85 ± 0.5 mCi/μmol

Supplemental Figure 3: Analytical HPLC of [1-^11^C]DHA t_R_ = 4.5 minutes

***Reduced glutathione (GSH) quantification assay***

*In vitro* GSH quantification assay was performed using a commercial available kit (Abcam, Cambridge, MA). Brain tissues were collected from rats and snap-froze in -80ºC immediately upon extraction. A non-fluorescent dye that becomes strongly fluorescent upon reacting with glutathione was added to the tissue supernatant after deproteinization (Abcam, Cambridge, MA), and the fluorescent signal was read by a microplate reader (Infinite M200, Tecan Group Ltd., Männedorf, Switzerland) at Ex/Em = 490/520 nm. In parallel, a BCA protein quantification assay (Abcam, Cambridge, MA) was performed using the same tissue supernatant before deproteinization, and GSH quantifications were normalized to their corresponding protein quantifications and reported in the unit of μmol/g_protein.

***2D Chemical Shift Imaging (CSI) data of hyperpolarized [1-^13^C]DHA (Figure 2)***

|  | VitC/DHA | VitC/(VitC+DHA) |
| --- | --- | --- |
| Animal 1 | 1.62 | 0.62 |
|  | 0.51 | 0.34 |
|  | 0.65 | 0.39 |
|  | 0.92 | 0.48 |
| Animal 2 | 0.29 | 0.23 |
|  | 0.37 | 0.27 |
|  | 0.47 | 0.32 |
|  | 0.47 | 0.32 |
| Animal 3 | 0.81 | 0.45 |
|  | 0.64 | 0.39 |
| Animal 4 | 1.32 | 0.57 |
|  | 2.28 | 0.70 |
| Animal 5 | 0.97 | 0.49 |
|  | 1.03 | 0.51 |
|  | 1.44 | 0.59 |
|  | 1.94 | 0.66 |
| Animal 6 | 2.28 | 0.70 |
|  | 1.27 | 0.56 |

**Table 1**. 2D Chemical Shift Imaging (CSI) with hyperpolarized [1-^13^C]DHA of 6 normal rats: 18 voxels that correspond to brain tissue were included in the analysis. Peak heights of DHA and VitC resonance were quantified on the magnitude spectra, and VitC/DHA and VitC/(DHA+VitC) were calculated for each voxel.

***1D slab dynamic MR spectroscopy data (Figure 3 and 4)***

|  | Apparent rate (k_app_) | | Kinetic rate (k) | |
| --- | --- | --- | --- | --- |
|  | Baseline | DEM-Tx | Baseline | DEM-Tx |
| Animal 7 | 9.13 | 8.23 | 16.91 | 9.672 |
| Animal 8 | 6.68 | 2.73 | 16.18 | 5.286 |
| Animal 9 | 5.70 | 3.01 | 10.37 | 7.724 |
| Animal 10 | 6.23 | 2.14 | 14.57 | 7.254 |

Unit: s^-1^

**Table 2**: Kinetic parameters for VitC production decreased significantly after DEM treatment: apparent VitC production rate by linear fitting (k_app_, scaled and normalized to VitC SNR) and kinetic rates (k) by kinetic modeling

| 100% dose | k_app_ | k | VitC SNR | 60% dose | k_app_ | k | VitC SNR |
| --- | --- | --- | --- | --- | --- | --- | --- |
| Animal 9 | 5.70 | 10.37 | 464.37 | Animal 12 | 4.18 | 10.71 | 499.6615 |
| Animal 10 | 6.23 | 14.57 | 638.71 | Animal 13 | 3.28 | 7.83 | 383.6483 |
| Animal 11 | 2.88 | 8.72 | 427.87 | Animal 14 | 5.67 | 14.27 | 639.4243 |

**Table 3**: Kinetic parameters and sum signal-to-noise ratio (SNR) for [1-^13^C]VitC remain unchanged with 60% dose of [1-^13^C]DHA
